# When artificial intelligence becomes a job resource: how psychological capital fuels innovation in algorithm-driven workplaces

**DOI:** 10.3389/fpsyg.2026.1740508

**Published:** 2026-05-08

**Authors:** Yanru Liu, Qing Tian, Jiachun Chen

**Affiliations:** School of Business, Macau University of Science & Technology, Taipa, Macao SAR, China

**Keywords:** AI adoption, artificial intelligence (AI), error management culture, innovative work behavior, psychological capital

## Abstract

**Introduction:**

This paper examines the relationship between AI adoption and employees’ innovative work behavior (IWB), focusing on the mediating role of psychological capital and the moderating role of perceived error management culture.

**Methods:**

Using two-wave survey data, we tested the hypothesized relationships with SPSS 27.0 and Mplus 8.0.

**Results:**

The results show that AI adoption is positively related to employees’ psychological capital, which in turn is positively related to IWB. In addition, perceived error management culture strengthens the positive relationship between AI adoption and psychological capital, thereby strengthening the indirect relationship between AI adoption and IWB through psychological capital.

**Discussion:**

By conceptualizing AI as a new form of job resource, this study examines the internal mechanism and boundary condition underlying the AI-innovation link through a “technology-psychology-behavior” framework. Theoretically, we extend JD-R theory to the AI context and incorporate error management culture through trait activation theory. In practice, we provide empirical evidence on how AI adoption is associated with employee innovation through psychological capital.

## 1 Introduction

In today’s unpredictable business environment, characterized by BANI (*Brittle, anxious, non-linear, uncomprehensible*), employee creativity for sustainable innovation is important and challenging for organizations. In this context, AI is emerging as a defining force that changes work behavior and accelerates knowledge competition (Wang et al., 2023; Alexy et al., 2013). Over 50% of businesses use AI to support knowledge creation, standardization, transfer, and thus increase their competitive edge (Iaia et al., 2023). According to McKinsey, 72% of the 1,636 companies have incorporated AI into their operations (generative AI adoption is increasing from 33 to 65% annually). This rapid digitalization requires employees to adapt to digital and intelligent work structures. However, existing studies focus on technology implementation, ignoring psychological mechanisms and context-specificities of AI-driven organizational transformation (Bankins et al., 2024).

One of the most important studies in organizational psychology examines how AI affects employee innovation work behavior (IWB) in changing technological settings (Belanche et al., 2019). IWB refers to employees creating creative ideas for products, processes, or procedures at work and trying to implement them to improve organizational efficiency (De Spiegelaere et al., 2014). AI can theoretically help employees innovate work behavior (IWB) by addressing two kinds of barriers people face before creating new ideas (Eggers and Kaplan, 2009): (1) *information processing constraints* (Nelson and Winter, 1982; Williams and Mitchell, 2004; Marois and Ivanoff, 2005) where limited human cognitive abilities inhibit knowledge acquisition; (2) *ineffective or local search routines* (Levinthal and Gavetti, 2000; Katila and Ahuja, 2002) where employees depend too much on familiar knowledge domains, limiting breakthrough innovations (Posen et al., 2018). AI mitigates these barriers by enabling cross-domain knowledge integration and opportunity identification (McAfee and Brynjolfsson, 2016), helping employees overcome cognitive limitations and generate new ideas (Akter et al., 2022). This shows how AI is not merely a tool but a catalyst for cognitive liberation and the generation of creative ideas.

A theoretical tension persists: AI is both a stressor and an innovation catalyst. Efficient studies show that AI may lead to perceptions of technological threats and job insecurity, increasing stress (Chen et al., 2025). Additionally, algorithmic management systems weaken autonomy by monitoring, thereby increasing role stress and emotional exhaustion (Rai et al., 2019). Complexity can increase cognitive load (Parker and Grote, 2022a) and negative emotions such as anxiety (Song et al., 2024), which may hinder IWB (Chang et al., 2024). In contrast, AI can overcome human cognitive limitations and facilitate human–computer collaborations (Brynjolfsson and McAfee, 2017). Findings indicate (a) AI indirectly increases employee creativity through active learning and task crafting (Wang et al., 2022).

However, there are still many differences in the “substitution versus promotion” debates on two key points: (1) the mediators are understudied, especially psychological resources such as self-efficacy, and (2) boundary conditions, such as organizational culture, are understudied, which buffer or enhance AI stressors. To address these problems, we reposition AI tools as new work resources (JD-R) in the JD-R framework (Demerouti et al., 2001). We propose that AI can enhance employees’ IWB through psychological capital, such as self-efficacy and optimism, which is mediated by perceived control and cognitive flexibility (Xanthopoulou et al., 2009). Further, we introduce error management culture [practices that minimize errors while encouraging learning (Edmondson, 1999)] that moderates this pathway by buffering AI risks, such as fear of failure, and increasing resource gains through psychological safety and trait activation (Tett and Burnett, 2003). This model reconciles the dual-effect debate in AI (Dong et al., 2025) by showing how organizational contexts channel AI’s instrumental value into drivers of sustainable innovation.

Our contribution is in four areas. First, we improve the JD-R model by defining AI as “work resources” rather than as “technical pressure,” thus extending the model beyond physical resources or interpersonal resources. Second, we address the lack of empirical evidence on the internal processes and boundary conditions that affect employees’ IWB through psychological capital, and we introduce a new “technology-psychology-behavior” model. Third, Trait Activation Theory demonstrates how error management culture interacts with individual resources (psychological capital) to facilitate technical adaptive behavior and provides a theoretical framework for research on the management of emerging technologies. Fourth, we provide both theoretical and practical guidance for managers to promote AI–human collaboration and emphasize the importance of proactive adaptation to digital transformation.

## 2 Theoretical background and hypotheses development

The Job Demands-Resources (JD-R) model (Demerouti et al., 2001) posits that workplace outcomes are driven by the interaction between job demands (physical, psychological, or organizational stress) and job resources (functional elements that reduce demands or support goal attainment) (Bakker and Demerouti, 2017). The dual-path mechanism has been widely validated: The high demands of the job erode energy and burnout (health impairment pathway). Traditional concepts of job resources generally focus on physical (e.g., ergonomic tools), social (e.g., supervisor support), and organizational (e.g., autonomy) (Bakker et al., 2023). However, the rapid integration of AI into workplaces demands a re-examination of the resource typologies in the digital age. Unlike previous studies that considered technology stressors (e.g., technostress from AI opacity), new evidence suggests that AI can serve as a novel cognitive resource that enhances human capabilities (Parker and Grote, 2022b). Conversational AI tools enhanced problem-solving efficiency by algorithms (Benbya et al., 2020). However, gaps remain: (1) limited empirical study of AI’s role in fulfilling JD-R motivation pathway—positive psychological traits and resources (PsyCap)—i.e. the individual’s positive psychological traits and resources and malleable state of self-efficacy, hope, resilience and optimism (Youssef and Luthans, 2007); (2) the boundary conditions, such as error management culture (Keith and Frese, 2008), that may modulate AI’s resource effects, are rarely studied.

This study aims to fill existing gaps by redefining AI as “intelligent cognitive resources” within the JD-R framework, thus broadening its theoretical application to human–AI collaboration contexts. Leveraging the resource gain spiral perspective (Hobfoll, 2011), we suggest that AI-enabled resources enhance self-efficacy through AI-assisted mastery experiences, which subsequently stimulate innovative behaviors. The overall model of this study is rooted in the JD-R theoretical framework, viewing AI tools as a type of work resource that offers ongoing decision support, a personalized learning platform, and a dynamic feedback mechanism. These AI tools are linked to enhanced employees’ perceived control over their environment and self-efficacy, are associated with the accumulation of psychological capital, which in turn encourages innovative behavior. This approach aligns with recent calls to incorporate technology affordances into JD-R’s motivational mechanisms (Scholze and Hecker, 2024).

### 2.1 The mediating role of psychological capital

The JD-R model (Demerouti et al., 2001) describes work environments as two kinds: job demands (negatives such as over-charges of energy) and job resources (positives such as goals, psychological costs, and personal growth). AI needs to be reconsidered in the digital age, considering it as a new kind of work resource. High job demands and low resources can lead to burnout, but only high resources can lead to engagement (Demerouti et al., 2001). Increasing job resources (such as autonomy and social support) can reduce burnout and encourage engagement. Psychological capital (Youssef and Luthans, 2007) was later incorporated into the JD-R model (Xanthopoulou et al., 2009), showing its importance in transforming resources into positive outcomes. Based on this, we define AI tools as new work resources in the JD-R model. Unlike previous studies which categorize AI as a “stressor” (Dong et al., 2025) or algorithmic surveillance (Rai et al., 2019), we highlight AI’s role in augmenting resources in three ways: (1) Cognitive Assistance where AI reduces knowledge acquisition barriers by intelligent retrieval systems, decreasing cognitive load in complex domains; (2) Emotional Buffering, in which AI’s impersonal interaction reduces evaluation anxiety in interpersonal communication and encourage open-ended problem exploration; (3) Task Optimization in which repetitive tasks free employees’ high-level cognitive resources to explore new practices; These functions help boost perceived control and self-efficiency, key psychological capital, which is associated with employees to challenge old practices and propose new solutions. In this study, AI adoption refers to employees’ perceived integration of AI tools into their daily work routines, including the frequency of AI use, reliance on AI for task completion, and the perceived relevance of AI to one’s job role (Man Tang et al., 2022). Based on the resource gain spiral theory (Hobfoll, 2011), we suggest that AI adoption, as a new work resource, is positively associated with employee innovation behavior, which in turn fuels innovation through intrinsic motivation (Amabile and Pratt, 2016) and cognitive flexibility (Yao and Li, 2023). From a technical point of view, we view AI tools as a supporting work resource that helps employees work more productively. From a psychological point of view, AI resources can reduce employees’ cognitive load by automating repetitive tasks and increase their mental resources for creative activities. By providing continuous decision support, learning tools, and feedback mechanisms, AI tools can be positively linked to employees’ perceived control over the environment and psychological capital.

Meanwhile, AI is also increasingly being used as a job resource in organizations, as stated in the Job Demands-Resources (JD-R) framework: Technology enhances employees’ ability to achieve innovation goals (Bakker and Demerouti, 2017). Empirical evidence indicates AI fosters innovative work behaviors (IWB) through two synergistic pathways: (1) cognitive augmentation—automating routine tasks to optimize human–AI symbiosis in complex decision-making (Brynjolfsson et al., 2014), and (2) psychological empowerment—reducing cognitive load and emotional exhaustion to reallocate mental resources toward creative problem-solving (Hu et al., 2023). These mechanisms reflect AI’s dual role as functional infrastructure (data-driven insights) and psychological enabler (e.g., workload reduction); however, the psychological processes by which AI-enabled resources are translated into sustained innovation remain unexplored. Based on positive organizational behavior literature, we argue that psychological capital (a malleable state comprising self-efficacy, hope, resilience, and optimism) is an important mediator (Youssef and Luthans, 2007). By reducing AI-induced cognitive demands and enhancing adaptive capacities, psychological capital may play a key mediating role in the gap between AI-driven cognitive demands and employees’ innovative outcomes, thereby filling a critical theoretical gap in JD-R-driven innovation research.

PsyCap plays a key role in innovation in the digital age, as it associated with enhanced individuals’ adaptability to technology and is linked to the availability of cognitive resources for creative problem-solving. Amabile and Pratt (2016) showed that psychological capital can stimulate innovation by motivating individuals (self-driving) and using cognitive flexibility (problem reformulation). Yao and Li (2023) found that digital technology stimulates innovation, and positive psychological capital helps entrepreneurs cope better with challenges. A quasi-experiment in business simulation games shows that AI support improves entrepreneurial attitudes; the study’s instrument also included psychological capital among the measured variables, indicating that AI-enabled learning contexts can shape motivational resources relevant to PsyCap (Chen et al., 2022).

Therefore, we believe that when employees view AI adoption as a work resource, it will increase their work engagement and psychological capital and help them generate innovative behavior. That is, from a technical point of view, AI adoption breaks through information processing limitations → enhances self-efficacy and hope; from an emotional point of view, AI reduces emotional exhaustion → enhances resilience and optimism. On this basis, we hypothesize:

*Hypothesis 1*: AI adoption is positively related to employees’ psychological capital.

We then explain why psychological capital positively related to employee innovation behavior. Innovative behavior is a complex, high-risk activity that involves considerable uncertainty. Employees may encounter various challenges and risks when carrying out innovative behaviors (He et al., 2019). Thus, stronger internal motivation is an important driver of innovative behaviors (Grant and Berry, 2011). According to the JD-R theory mentioned above, work resources have motivational potential, and promoting psychological capital is conducive to innovative behavior. Employees with higher psychological capital tend to cope more effectively with work challenges and solve problems more effectively, leading to new thinking and behavior. Luthans and Youssef-Morgan (2017) discussed psychological capital in relation to learning and organizational behavior. They pointed out that psychological capital can help employees learn more effectively, apply knowledge better, and develop innovative thinking and behavior. Self-confidence and hope motivate employees to actively seek new knowledge and skills, while optimism and resilience make them more positive and persistent in times of difficulty. Thus, we hypothesize:

*Hypothesis 2*: Psychological capital is positively related to employees’ innovative work behavior (IWB).

Integrating *Hypothesis 1* and *Hypothesis 2*, we propose the following hypothesis:

*Hypothesis 3*: Psychological capital mediates the relationship between AI adoption and employees’ innovative work behavior (IWB).

### 2.2 The moderating role of perceived error management culture

In particular, we extend the mediated pathway (AI → Psychological Capital → Innovative Work Behavior) by using error management culture as a moderator. Error management culture is defined as the error-related behaviors and actions within an organization that aim to minimize the negative effects and enhance the positive effects of errors (Edmondson, 1999). Error management culture enhances AI’s resource-enhancing effects through two mechanisms: Conservation of Resources (COR) theory (Hobfoll, 1989) and the Job Demands-Resources (JD-R) model (Demerouti et al., 2001). First, it buffers AI-induced psychological risks (e.g., fear of failure) by fostering a psychological safety environment, in which employees perceive mistakes as opportunities for growth rather than threats (Nerstad et al., 2018). Second, it synergizes with AI’s instrumental functions (e.g., task automation, cognitive aid) to create a resource gain spiral, boosting employees’ perceptions of control and self-efficacy (Xanthopoulou et al., 2009).

In a strong error management culture, resources such as error communication, error analysis, learning from errors, and error competence (effective error handling strategies) are available to help employees improve their performance, handle problems effectively, and accept help when needed.

According to Trait Activation Theory (Tett and Burnett, 2003), organizational culture serves as a situational cue that activates the expression of individual traits or resources by providing supportive environments. A culture of high error management creates psychological safety, enabling employees to adopt AI for innovative behavior by reducing the fear of failure and reframing misperceptions, such as viewing errors as learning opportunities (Edmondson, 1999). This safe environment maximizes the empowering potential of AI tools as work resources: employees are more willing to try advanced AI features (such as generative design and complex data analysis), and they increase self-efficacy and hope through successful experience (psychological capital dimension). Furthermore, Van Dyck et al. (2005) empirically demonstrated that error management culture primarily enhances employee learning during the technology adoption process (i.e., resource acquisition) rather than in knowledge application. AI adoption promotes innovative work behavior by enhancing employees’ psychological capital, and an error management culture as an organization-level factor can moderate this process.

Empirical studies underscore this dual role. For example, an experiment published in *Science* (Noy and Zhang, 2023) demonstrated that utilizing artificial intelligence technologies, such as ChatGPT, can help reduce the differences between individuals. Moreover, even though the AI-generated drafts sometimes contained errors, the final output of the experimenters was significantly better than that of the control group without AI. Thus, AI tools like ChatGPT can improve people’s baseline cognitive performance, but it depends on cultures that tolerate experimentation. Trait activation theory (Tett and Burnett, 2003) shows that error management culture activates employees’ innovative traits by cultural signals (e.g., rewards for exploratory behavior) and increases the AI → Psychological Capital link. Error management culture moderates this pathway by: (1) reducing anxiety about AI-driven mistakes (which increases people’s engagement with AI tools and builds psychological capital); (2) strengthening synergy (e.g., reduced evaluation anxiety) and maintaining a resource gain spiral that fuels innovation.

This moderated mediation framework eases the AI technology “double-edged sword” debate a bit by showing how organizational culture channels AI risk into innovation drivers. By setting error management culture as a boundary condition, we provide practical ways to harness AI’s power while avoiding its paradoxical consequences.

On the one hand, an error management culture is a supportive work environment that fosters strong fault tolerance and correction mechanisms and provides employees with greater psychological security. Using AI techniques like ChatGPT can help narrow the differences between people who use AI to complete analytical tasks. However, AI can be misleading, as its quality score is relatively high even when it makes mistakes (Noy and Zhang, 2023). Therefore, in an error management culture, employees can reduce their worry about using AI to make mistakes and face ridicule and punishment (Cheng et al., 2022), and gain confidence and support to take risks. On the other hand, high level error management culture is also conducive to communication, mutual help and learning among team members after errors are made (Maurer et al., 2017). AI tools also improve employees’ ability, technology, and self-confidence. Employees can invest in creative activities to build higher levels of psychological capital, grasp organizational requirements and market needs, and contribute to innovative work behavior.

This paper operationalizes perceived error management culture (PEMC) as the primary explanatory variable, rather than relying on objective organizational culture measures. This decision follows two main reasons:

Methodically, the online longitudinal survey design (e.g., via the Credamo platform) required cross-industry sampling, which posed difficulties with multi-source aggregated data (e.g., multi-respondent organizational samples) or records (e.g., internal policy documents). Moreover, as the question concerns individual-level micro-mechanics (e.g., innovative behaviors), maintaining construct-level consistency across variables was needed to avoid ecological mistakes (Klein and Kozlowski, 2000).

Theoretically, individual behaviors are driven by subjective interpretation of environmental cues (Bandura, 1986). Employee perceptions of organizational error tolerance (i.e., whether leaders encourage error-based learning) directly shape psychological safety and risk-taking behavior (Keith and Frese, 2008). Based on the above analysis, we thus propose that:

*Hypothesis 4*: Perceived error management culture positively moderates the relationship between AI adoption and psychological capital, such that the relationship will be stronger at a higher level of perceived error management culture rather than at a lower level.

**Figure 1:**
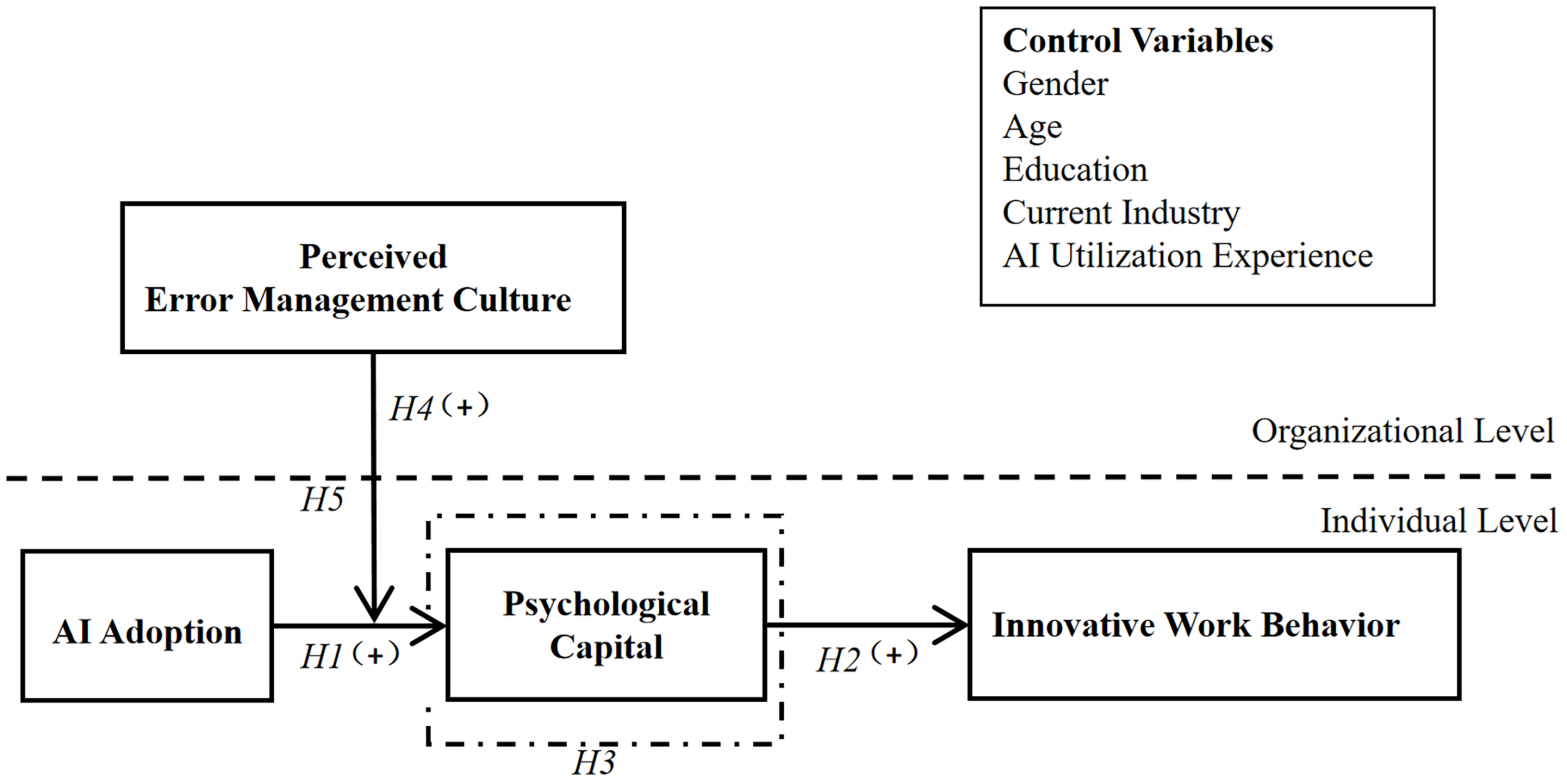
Theoretical framework.

Therefore, by combining *Hypotheses 3* and 4, we further propose the moderated mediation hypothesis:

*Hypothesis 5*: Perceived error management culture will moderate the indirect relationship of AI adoption on employees’ IWB through psychological capital, such that the indirect relationship will be stronger at a higher level of perceived error management culture rather than at a lower level. The theoretical model of this study is presented in Figure 1.

## 3 Methods

### 3.1 Participants and data collection

This study employed a two-wave, non-probability purposive sampling strategy. We primarily utilized Credamo, an online survey platform, to gather data by recruiting participants from its panel pool (i.e., the data mart) for a two-wave follow-up survey. As this study focused on employees with actual workplace exposure to AI, we administered screening questions before the formal survey. Participants were first asked, “Do you use AI regularly in your work?” If they answered ‘No,’ the questionnaire was automatically terminated. They were also asked, “How long have you been exposed to and used AI tools?” and “How often do you interact with and use AI tools?” In addition, we restricted the target sample to industries and organizational contexts in which AI tools were more likely to be used in daily work, while excluding labor-intensive industries or work settings with very limited AI exposure. This purposive screening procedure ensured that the sample was relevant to the study purpose.

To reduce common method bias (CMB), we adopted the two-wave questionnaire collection method recommended by Podsakoff et al. (2003), with a 20-day interval between the two waves. In the first phase, we collected information on AI adoption, perceived error management culture, and participants’ demographic characteristics. Through Credamo’s data mart, 608 participants were recruited and invited to complete the first-wave survey. After eliminating responses from individuals who provided overly brief answers or did not complete the survey seriously, 520 valid questionnaires were retained, yielding an effective response rate of 85.53%. In the second phase, respondents who completed the first-wave survey were invited to participate in a follow-up survey assessing employees’ psychological capital and innovative work behavior. Importantly, Credamo’s follow-up survey function generated a unique anonymous tracking code for each participant who completed the first-wave questionnaire, and the second-wave questionnaire was pushed directly to the same respondents through the platform app and its affiliated WeChat channels. The platform’s built-in matching function was then used to automatically link the two waves. This procedure allowed longitudinal matching while preserving anonymity and confidentiality and reducing potential matching errors associated with manual processing. A total of 520 s-wave questionnaires were distributed, and 434 were recovered, yielding an effective response rate of 83.46%.

At the same time, we supplemented the online sample with a field sample collected from one cooperating enterprise through an intermediary contact person (the board secretary). The intermediary assisted in distributing the questionnaires in two stages to employees from multiple departments, including finance, human resources, and production. The same eligibility criteria were applied to the enterprise sample. For matching across the two waves, respondents in the enterprise sample provided only the last four digits of their mobile phone numbers for anonymous matching purposes. In total, 32 questionnaires were collected from the enterprise sample. This procedure enabled us to match the two waves while minimizing ethical risk and protecting participant privacy.

After eliminating questionnaires with apparently regular response patterns and failed matches, 449 valid matched questionnaires were obtained. In the valid sample, 41.4% of respondents were male, and 58.6% were female, and the average age was 31 years. In terms of education, 29 respondents (6.5%) had a college degree or below, 307 (68.4%) held a bachelor’s degree, and 113 (25.2%) held a master’s degree or above. In terms of industry distribution, 33.2% worked in manufacturing, 25.4% in software and information technology services, 18.0% in education, 9.1% in finance, and 3.8% in scientific research and technology services.

### 3.2 Measurement

The scales used in this study are mature scales that have been used at home and abroad. To ensure the scales’ scientificity and accuracy, measures were translated into participants’ native languages using recommended back-translation procedures. (1) *AI adoption*: We adopt the scale compiled by Man Tang et al. (2022), which contains three items, such as “I spent most of the time working with artificial intelligence”. The Cronbach’s alpha coefficient for this scale is 0.827. (2) *Psychological capital*: We adopt Luthans et al. (2007), which consists of 4 dimensions (*Self-Effectiveness, Hope, Resilience, Optimism*) and a total of 24 items. Typical topics include “I believe I can analyze long-term problems and find solutions”. The Cronbach’s *α* coefficient of this scale is 0.868. (3) *Innovative work behavior*: We adopt Ng and Lucianetti (2016) scale, which contains nine items, such as “I search out new working methods, techniques, or instruments”. Cronbach’s alpha coefficient is between 0.79 and 0.87. (4) *Perceived error management culture*: To measure employees’ subjective interpretations of organizational error management practices, we adapted the 17-item Error Management Culture scale initially developed by Deng et al. (2017) based on Van Dyck et al. (2005). The original scale, designed to assess organizational-level cultural norms (e.g., “In this organization, errors lead to improved processes”), was reframed to capture individual-level perceptions by phrasing items in the first person (e.g., “I believe that errors in my organization can effectively improve our workflow”). The Cronbach’s *α* coefficient of this scale is 0.912. All items of the scale were scored on a 5-point Likert scale ranging from 1 = “strongly disagree” to 5 = “strongly agree”.

In addition, to control for potential confounding factors, demographic variables such as gender, age, industry type, educational background, and length of AI use were included.

### 3.3 Data analysis

We performed confirmatory factor analysis and common-method bias testing, and conducted path analysis using Mplus 8.0. We used SPSS 27.0 for Harman test, descriptive statistics, correlation analysis, and hierarchical regression analysis. We tested the mediating role of psychological capital using bootstrapping to estimate confidence intervals. We also tested first-stage indirect relationship and differences between high-moderating and low-moderating variables.

## 4 Results

### 4.1 Descriptive statistical analysis

Table 1 summarizes descriptive statistics and zero-order correlations. To test discriminant validity, we examined the cross-loadings of indicators and the loadings of each indicator. All indicators had loadings above 0.70 on their respective constructs, and cross-loadings on other constructs were below 0.50, indicating clear differentiation between the latent variables. The correlation matrix has no high correlations (all *r* < 0.90), alleviating concerns about multicollinearity (Kock and Lynn, 2012). Although AVE-based Fornell-Larcker criteria were not computed due to scale adaptation constraints, the observed pattern of correlations (e.g., *r* = 0.59 between PEMC and PC) corresponds to theoretical expectations and warrants further structural analysis. AI adoption is positively correlated to psychological capital (*r* = 0.276, *p* < 0.01); psychological capital is positively correlated with innovative work behavior (*r* = 0.888, *p* < 0.01); and AI adoption was positively correlated with employee innovative work behavior (*r* = 0.216, *p* < 0.01). These findings provide preliminary support for the hypothesized relationships.

**Table 1:** Descriptive statistics and zero-order correlations (*N* = 449).

Variables	Mean	Standard deviation	*α*	1	2	3	4	5	6	7	8
1 Gender	1.59	0.49									
2 Age	2.13	0.77		−0.08							
3 Education	3.19	0.61		0.10^*^	−0.11^*^						
4 Current industry	8.83	5.01		0.05	−0.40^**^	0.03					
5 AI utilization	3.06	0.79		−0.04	−0.07	−0.02	−0.08				
6 AI	3.98	0.72	0.77	−0.09	0.06	−0.04	−0.19^**^	0.25^**^			
7 PC	4.20	0.45	0.92	0.03	−0.08	0.20^**^	−0.02	0.20^**^	0.28^**^		
8 PEMC	4.29	0.29	0.72	−0.02	0.01	0.07	−0.08	0.15^**^	0.36^**^	0.60^**^	
9 IWB	4.17	0.57	0.87	0.05	−0.09	0.19^**^	−0.03	0.20^**^	0.22^**^	0.89^**^	0.52^**^

^***^p < 0.001, ^**^p < 0.01, ^*^p < 0.05.

### 4.2 Confirmatory factor analysis

Before testing hypotheses, we conducted a confirmatory factor analysis (CFA) to test the discriminant validity of four potential variables: AI adoption, psychological capital, error management culture, and innovative work behavior. The model’s fit is evaluated using the *χ*^2^/df ratio, CFI, TLI, RMSEA, and SRMR. Table 2 shows that the four-factor model provides the best fit (*χ*^2^ = 2057.569, df = 813, *χ*^2^/df = 2.531^***^, RMSEA = 0.058, SRMR = 0.055), and all indicators are better than those of the other three-factor models. This indicates that the four-factor model fits well and discriminates well between the primary variables in this study (see Table 2).

**Table 2:** The confirmatory factor analysis.

Model	*χ* ^2^	df	*χ*^2^/df	CFI	TLI	RMSEA	SRMR
Hypothesized 4-factor model	2057.57	813	2.53^***^	0.83	0.82	0.058	0.055
3-Factor model 1 (AI + PC)	2261.96	816	2.77^***^	0.81	0.80	0.06	0.06
3-Factor model 2 (AI + IWB)	2266.29	816	2.78^***^	0.81	0.80	0.06	0.06
3-Factor model 3 (PC + IWB)	2180.10	816	2.67^***^	0.83	0.82	0.06	0.06
3-Factor model 4 (PEMC + IWB)	2148.24	816	2.63^***^	0.82	0.81	0.06	0.06
1-Factor model (AI + PC + PEMC + IWB)	2348.98	819	2.87^***^	0.80	0.79	0.07	0.06

***p < 0.001, **p < 0.01, *p < 0.05. *N* = 449; AI represents AI adoption, PC represents psychological capital, IWB represents innovative work behavior, PEMC represents perceived error management culture; + represents consolidation into a factor.

### 4.3 Common method bias

To mitigate the impact of common method bias (CMB) on the findings, we randomized the item order during measurement and emphasized the confidentiality of the research. Three methods were used to test the CMB issue. Firstly, we conducted Harman’s single-factor test, the results showed that eight factors with eigenroots greater than one were obtained without rotation, and the variance explained by the first factor was 32.238% (<40%) (Podsakoff and Organ, 1986), so the problem of common method bias was not serious. Second, we constructed a one-factor model by placing all items into a single factor, as shown in Table 2. The model fit was poor (*χ*^2^ = 2348.977, df = 819, CFI = 0.795, TLI = 0.785, RMSEA = 0.065, SRMR = 0.058), and significantly lower than the benchmark model. Finally, according to the common latent factor (CLF) approach (Podsakoff et al., 2003), we constructed a CLF model by adding a common factor to the benchmark model. Although the CLF model fit the data equally well (*χ*^2^/df = 2.258, CFI = 0.869, TLI = 0.855, RMSEA = 0.053, SRMR = 0.049), the addition of the common factor did not improve the benchmark model (Δ*χ*^2^/df = 0.273, ΔCFI = 0.036, ΔTLI = 0.031, ΔRMSEA = 0.005, ΔSRMR = 0.006). In summary, the CLF analysis did not show any effect of common method bias (ΔCFI = 0.036); future work should check if conclusions are robust by using multi-source data.

To assess the impact of CMB, we used the unmeasured latent method factor (ULMC) model. The results showed that the normalized load squares of method factor pairs were all less than 20% (M = 15.3%, SD = 4.1%), and the critical path coefficients ranged from −6.3% to −8.9% (all <10%). Although ΔCFI = 0.036 and ΔTLI = 0.031 in the CLF analysis were slightly above the recommended thresholds (recommended <0.02), combined with the ULMC results and path stability, this study concluded that common method bias did not substantially alter the conclusions. Future studies can further control for potential bias by using multi-wave data collection.

### 4.4 Hypothesis testing

#### 4.4.1 Mediating effect test

As shown in Table 3, AI adoption was positively related to employees’ psychological capital (Model 1: *β* = 0.129, *p* < 0.001), supporting *Hypothesis 1*. Psychological capital positively predicted IWB (Model 2: *β* = 1.104, *p* < 0.001; Model 4: *β* = 1.104, *p* < 0.001), supporting *Hypothesis 2*. Furthermore, the indirect correlation of AI adoption on IWB via psychological capital was significant, indicating that psychological capital mediated the relationship between AI adoption and IWB, thereby supporting *Hypothesis 3*.

**Table 3:** Unstandardized estimates (standard error) for the moderated mediation model.

Variables	Simple mediation model (PC)		Moderated mediation model (IWB)	
	Model 1	Model 2	Model 3	Model 4
Control variables				
Gender	0.04 (0.04)	−0.00 (0.03)	0.03 (0.03)	−0.00 (0.03)
Age	−0.04 (0.04)	−0.01 (0.02)	−0.02 (0.03)	−0.01 (0.02)
Education	0.16^***^ (0.04)	−0.00 (0.02)	0.09^***^ (0.03)	−0.00 (0.02)
Current industry	0.00 (0.00)	−0.01 (0.00)	0.00 (0.00)	−0.01 (0.00)
AI utilization experience	0.08^**^ (0.03)	0.02 (0.02)	0.05^*^ (0.03)	0.02 (0.02)
Independent variable				
AI adoption	0.13^***^ (0.02)	−0.01 (0.02)	0.05^*^ (0.02)	−0.01 (0.02)
Mediator variable				
Psychological capital		1.10^***^ (0.04)		1.10^***^ (0.04)
Moderator variable				
Perceived error management culture			0.29^***^ (0.03)	
Interaction				
AI adoption × perceived error management culture			0.06^**^ (0.02)	
*R*^2^	0.14	0.78	0.49	0.80

*N* = 449; ^***^p < 0.001, ^**^p < 0.01, ^*^p < 0.05.

To further confirm the significance of this mediation relationship, we conducted a 5,000-replicate bootstrap test (Preacher and Hayes, 2008). The results of the indirect relationship analysis are shown in Table 4. Bootstrap mediation analysis (*N* = 449) showed that AI adoption had a significant indirect correlation on employee innovation work behavior (IWB) via psychological capital (PC) [*β* = 0.143, 95% CI = (0.093, 0.197)] and a significant total relationship (coefficient = 0.131, *p* < 0.001). However, the direct relationship was insignificant (*β* = −0.012, *p* = 0.467), suggesting that psychological capital plays a mediating role in the relationship between AI adoption and innovation work behavior. This result further supports *Hypothesis 3*. However, the variation in the main relationship is explained by the moderating variable.

**Table 4:** Results of bootstrap testing for the mediation and moderation models.

Effect	Coefficients	S.E.	95% CI LL	95% CI UL	*p*
Total estimate	0.13	0.03	0.07	0.19	0.00
Direct estimate	−0.01	0.02	−0.04	0.02	0.47
Indirect estimate	0.14	0.03	0.09	0.20	0.00
High moderator (PC → IWB)	0.12	0.03	0.07	0.18	0.00
Low moderator (PC → IWB)	−0.01	0.04	−0.08	0.02	0.34
Difference (high vs. low)	0.13	0.04	0.04	0.22	0.00

*N* = 449; S.E., standard error; CI, confidence interval; LL, lower limit; UL, upper limit.

#### 4.4.2 Moderating effect test

**Figure 2:**
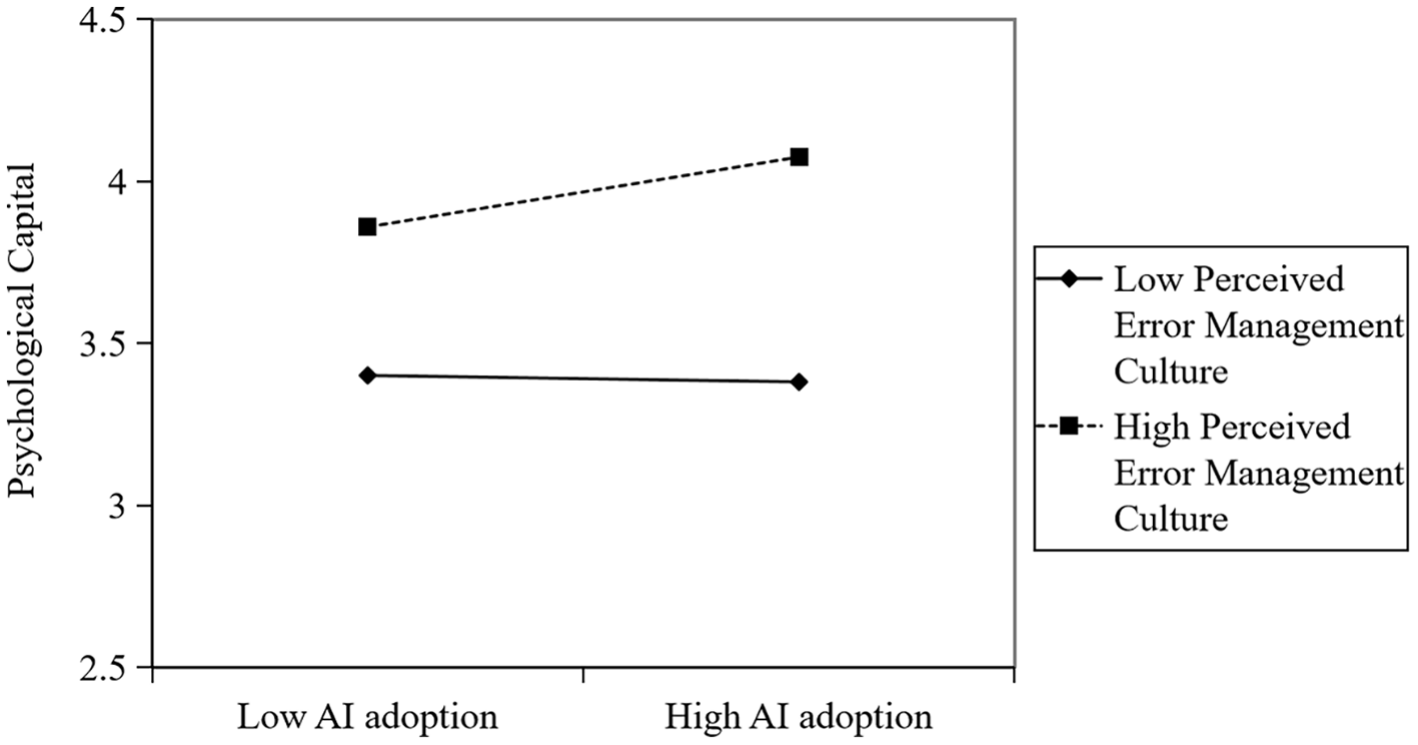
The moderating effect of perceived error management culture on AI adoption and psychological capital.

The interaction between AI adoption and perceived error management culture was significant (*β* = 0.059, *p* < 0.01), and that perceived error management culture moderated the relationship between AI adoption and psychological capital (see Table 3). Simple-slope analysis further showed that the positive relationship between AI adoption and psychological capital was significant. As shown in Figure 2, when the perceived error management culture is high (M + 1 SD), the positive relationship between AI adoption and psychological capital is significant (simple slope = 0.119, *t* = 4.421, *p* < 0.001). However, when the perceived error management culture was low (M − 1 SD), there was no significant relationship between AI adoption and psychological capital (simple slope = −0.011, *t* = −0.957, *p* > 0.05). These results indicate that perceived error management culture strengthened the positive relationship between AI adoption and psychological capital. Therefore, *Hypothesis 4* was supported.

#### 4.4.3 Moderated mediation test

As shown in Table 4, in the mediating path of psychological capital, when the perceived error management culture is strong (M + 1SD), the indirect relationship estimate of AI adoption on employees’ innovative work behavior through psychological capital is 0.119, and the 95% confidence interval is [0.071, 0.178], excluding 0; When the perceived error management culture is weak (M − 1SD), the indirect relationship estimate of AI adoption on employees’ innovative work behavior through psychological capital is −0.011, and the 95% confidence interval is [−0.075, 0.016], including 0. The difference between the high and low indirect relationship estimate was 0.130, with a 95% confidence interval of [0.043, 0.217], excluding 0, indicating significant differences. Thus, *Hypothesis 5* is supported.

## 5 Discussion

Based on the JD-R model and trait activation theory, we believe that AI adoption can be regarded as a form of technical support and work resource enriching employees’ psychological capital, which provides positive possibility for research background and empirical results; perceived error management culture positively moderates the relationship between AI adoption and psychological capital by providing supportive environment to activate individual trait or resource expression. Our hypotheses are confirmed by empirical testing of the relationships between these variables. To sum up, JD-R and Trait Activation Theory provide a solid theoretical basis for our research and can effectively explain individual employees’ positive responses to emerging technologies. In addition, this study provides new insights and ideas for organizations to seek employee cooperation and support in introducing new technologies or advancing intelligent transformation.

First, we consider AI as a job resource, building on and expanding the JD-R theory. We agree with recent studies on technology as a job resource, but we view AI adoption as a positive enabler rather than a stressor. While previous studies often referred to AI as two-edged (automation anxiety), our results show that it is positively associated with employees’ psychological resources. The positive link between AI and IWB is similar to that posited by, who found that digital tools are associated with creativity. We focus on AI, but we extend this work by emphasising its role in stimulating innovation through resource accumulation. This is a bridge between technology adoption studies and JD-R studies and supports the implementation of AI in the “resource caravan” (Hobfoll, 2011). Nevertheless, we acknowledge that while our findings emphasize AI’s positive role as a job resource, the JD-R framework also recognizes technology as a potential demand. Employees’ perceptions of AI may vary depending on individual and contextual factors. Future research could further explore conditions under which AI’s resource-enhancing potential is less pronounced.

Second, we found how AI influenced employee innovation by using psychological capital (PsyCap). We found that PsyCap mediates situational resources and latent traits, and the indirect relationship estimate is 0.143 (*p* < 0.001). This supports Trait Activation Theory, suggesting that situational resources activate latent traits. AI adoption of job resources increases employee self-efficacy, optimism, and resilience, key PsyCap components that fuel IWB. This is in line with Luthans et al. (2007), who highlighted PsyCap’s role in innovation, but we specifically identify AI as a catalyst. This mediation pathway highlights resource-driven agency: AI empowers employee to use their psychological resources to create creative outcomes rather than directly generating innovation.

Finally, we demonstrate that perceived error management culture (PEMC) modifies AI adoption and employee mental capital, thus increasing employee innovation. The moderating role of PEMC provides a key nuance to our model, acting as a boundary condition. In organizations with strong error management cultures, the correlation between AI adoption and psychological capital (PsyCap) was stronger (*β* = 0.48 vs. 0.29 in low-PEMC contexts). This supports Frese and Keith's (2015) hypothesis that supportive cultures maximize resource use. However, our results show how perceived EMC activates AI’s potential as a resource. By describing errors as learning opportunities, perceived EMC reduces perceived risks associated with AI-driven experiments, thus boosting PsyCap. This also extends the JD-R model by focusing on organizational culture as a contextual amplifier of resource-based relationships, an area that has not been previously explored.

## 6 Conclusion

This paper investigated the link between AI adoption and employee innovative work behavior (IWB). AI is a disruptive, knowledge-based technology that stimulates business development and is increasingly being studied by enterprises. AI adoption is promising, but most people remain skeptical, especially those who fear job loss to AI. This negative attitude often overlooks the benefits of AI–human interaction, which can help employee psychological capital and promote new work behavior. AI adoption is also a technical support and a job resource that can help employee psychological capital, and a fertile ground for further research and experiments. Core technology is key for enterprises, and the challenge requires independent innovation. Innovation is crucial for businesses to grow and sustain a competitive edge. We have developed a theoretical model to study how AI adoption is related to employee innovative work behavior (IWB). The results reveal that (1) AI adoption was positively related to employees’ psychological capital; (2) psychological capital fully mediated the relationship between AI adoption and innovative work behavior, with an indirect relationship estimate of 0.143 and a 95% confidence interval of [0.093, 0.197]; and (3) perceived error management culture moderated the relationship between AI adoption and psychological capital. When perceived error management culture was high (+1 SD), the positive relationship between AI adoption and psychological capital was stronger (simple slope = 0.119, 95% confidence interval [0.071, 0.178]). In addition, perceived error management culture strengthened the indirect relationship between AI adoption and innovative work behavior through psychological capital.

### 6.1 Theoretical implications

In this paper, we propose and prove the positive role of AI as a work resource. In contrast to previous work, AI was perceived as a stressor; we propose a positive psychology approach that validates AI’s role as a resource enabler. We provide a balanced theory of AI impact. We show that AI can be a resource-accreting force whose outcomes depend on the interaction between organizational context and individual cognition. AI is associated with increased employee psychological capital, such as self-efficacy, hope, and resilience, which is in turn related to positive and innovative work behavior. We challenge the pessimistic assumption of AI threat theory that AI technology is directly linked to employees’ innovative work behavior through a resource-gain path.

Second, using the JD-R theory, we develop a mediating model to study how AI is adopted in employees’ innovative work behavior, with the mediating role of psychological capital. AI adoption is associated with innovation through its relationship with employee psychological resources, such as cognitive barriers and work efficiency. In line with the JD-R theory notion of a “resource-gain spiral,” we incorporate the motivation path to show how AI can create a positive feedback loop via psychological capital accumulation. This addresses a gap in current work regarding AI’s association with innovation behavior by transforming mental resources.

Finally, we introduce the “perceived error management culture” as a moderator at the organization level, considering the limitations of individual moderators and checking its moderating role. This extends the boundary conditions of AI adoption on employees’ innovative work behavior and enriches empirical work on error management culture as a moderator. We find that a high perceived error-management culture, as a supportive work environment, is positively associated with employees’ psychological capital in the context of AI adoption and is linked to a greater investment of their individual psychological resources in innovative work behavior (IWB). When perceived error management culture is strong, indirect relationships are strengthened. We support Trait Activation Theory, which posits that supportive environments can transform individual resources into behaviors. A highly inclusive culture encourages employees to invest psychological capital in innovative work by decreasing their risk perception.

By revealing the mediating path of AI adoption → psychological capital →IWB and the moderating role of error management culture, we combine JD-R theory (resource-behavior path) with trait activation theory (environment-trait interaction) to construct a cross-layer model showing how individual resources (psychological capital) and organizational context (error management culture) drive technological adaptive behavior. We also define the synergy between AI and employees. Contrary to the narrative of “human–machine substitution,” we emphasize the possibility of “human–machine symbiosis” in the sense that AI adoption can boost overall organizational performance by increasing employee mental capital and stimulating the potential for innovation (e.g., autonomous learning, team creativity). This provides a theoretical basis for the intelligent transformation of enterprises, where the key to the successful implementation of AI lies in the collaborative optimization of technology and human values.

### 6.2 Practical implications

Our findings provide actionable insights for organizations seeking to leverage AI technology as a catalyst for innovation while promoting employee well-being and resilience.

First, AI can search and process vast amounts of information more efficiently than humans and store knowledge, which can help people identify problems, opportunities, and threats beyond local search routines and knowledge domains, and generate new ideas. Therefore, enterprises and employees can significantly improve their knowledge management capabilities through AI (Iaia et al., 2023). For example, AI can accelerate knowledge retrieval, improve the accuracy of knowledge organization and integration, and provide ideas and frameworks for structuring and systematizing knowledge. Therefore, adopting artificial intelligence can effectively promote knowledge management and innovation. It can be seen that enterprises’ introduction of intelligent technology should not be aimed at responding to the state’s call or at being forced to participate in social change. Individual employees or managers should rationally recognize AI’s significance for their own and the enterprise’s development, actively embrace emerging technologies, and promote the intelligent growth of organizations.

Second, businesses should train employees to learn skills rather than knowledge. Our study shows a positive correlation between AI adoption and psychological capital (PsyCap). Employees who see AI as a tool to improve their abilities rather than a threat are more self-motivated, optimistic, and resilient. Training programs should explain AI tools, such as how they can speed up knowledge retrieval, improve problem-solving accuracy, and stimulate creativity, for example, by brainstorming with generative AI. Companies should encourage employees to work with AI on real problems, indicating that AI complements human intelligence rather than replaces it. Positioning AI as a skill enhancer allows businesses to change employee perceptions from viewing AI as a disruptor to an enabler.

Thirdly, enterprises should implement fault tolerance policies, experiment with learning from failures, and establish a framework that supports innovation. These measures include: (1) updating performance metrics from outcome-based assessments to process-oriented evaluations encouraging exploration of AI even if the results are not optimal; (2) promoting transparency through empowering managers to share trial-and-error experiences with AI adoption demonstrating mistakes are growth opportunities; and (3) creating AI “sandbox” environments (low risk spaces like cross-departmental innovation labs) which allow employees to experiment with AI applications without fear of negative consequences. This approach aligns with trait activation theory as supportive environments unlock employee psychological resources and enable employees to transition from technology adaptation to innovation driven behavior. With the AI–human collaboration model, enterprises should maximize the enabling potential of AI by encouraging an open error management culture, providing empirical insights for managers to design AI training, optimizing the organizational support environment, and helping employees transition from “technical adaptation” to “innovation drive.”

Finally, AI is increasingly being integrated into leadership practices, showing how leaders shape perceptions and applications of AI. Our research shows that AI benefits the most when employees see it as a “work resource” rather than a threat to their jobs. Leaders should promote AI’s complementary function, emphasising its ability to increase human strengths. AI can handle data-intensive tasks, allowing employees to focus on creative tasks. Leaders should address ethical and occupational safety concerns by engaging in transparent discussions to reduce employee anxiety and emphasize AI’s role in skill enhancement rather than job replacement. By integrating skill-focused AI training, fault-tolerant policies, and a collaborative AI leadership approach, organizations can turn AI applications into sustainable innovation drivers, ultimately creating a culture where human creativity and AI can be combined.

### 6.3 Limitations and future research directions

While we have improved our understanding of Al′s role in workplace psychology, there are limitations on future work. (1) Time range of data: Although our two-wave survey design reduces common method bias, the limited time frame limits our understanding of long-term dynamics. For example, PsyCap may evolve as human–AI interaction matures, but it cannot be fully captured or understood. Future multi-wave studies could investigate how the adoption of AI affects mental capital and innovation at different stages of integration. (2) Causal Inference: The cross-sectional and non-experimental design of this study limits our ability to draw causal inferences. While our hypotheses, grounded in theory, proposed directional relationships, the correlational nature of our data means we can only confirm associations, not causality. It is possible that employees with higher psychological capital are simply more likely to perceive AI adoption positively (reverse causality), or that an unmeasured third variable influences both. Future research employing longitudinal or experimental designs is necessary to establish causal directions. (3) Cultural universality: Our samples come from a single culture and future studies should consider how differences in moderating effect differs in multicultural settings. The moderating role of error management culture and the relationship between AI and mental abilities might differ across cultures because different cultures have different attitudes toward technology and tolerance of error. Replicating the results in different cultures could clarify boundary conditions and enhance theoretical application. (4) AI typology: Our study focused on AI tools broadly defined (e.g., generative AI and large language models) as perceived by employees. Different AI technologies may cause different psychological and behavioral responses. For example, generative AI may improve creativity more directly than automated workflow tools. Future research should categorize AI subtypes to explore their varying associations with PsyCap and innovation. (5) Potential discriminant validity concerns: The observed zero-order correlation between psychological capital (PC) and innovative work behavior (IWB) is relatively high (*r* = 0.888, *p* < 0.01), which may raise concerns about construct redundancy. We acknowledge this issue and offer the following clarifications based on the results already reported in this manuscript. First, as shown in Table 2, the four-factor model (AI, PC, PEMC, IWB) fits the data significantly better than any alternative three-factor model (e.g., combining PC and IWB into one factor), and all indicator loadings exceeded 0.70 on their respective constructs with cross-loadings below 0.50. These results support that the four constructs are empirically distinguishable at the measurement level, even though they are strongly correlated. Second, a strong positive relationship between psychological capital and innovative work behavior is theoretically expected. Psychological capital—comprising self-efficacy, hope, resilience, and optimism—has been consistently shown to be a robust predictor of proactive and innovative behaviors (Luthans et al., 2007). Thus, a high correlation is not necessarily indicative of complete construct overlap but may partly reflect a genuine empirical relationship. Third, common method bias (CMB) may have inflated the observed correlation. As reported in Section 4.3, our CMB tests indicated that an unmeasured latent method factor accounted for approximately 15.3% of the total variance, and critical path coefficients changed by less than 10% after controlling for the method factor. This suggests that while CMB exists, it does not fully explain the relationship. Nevertheless, we concede that the high PC-IWB correlation should be interpreted with caution. Given these considerations, the mediation effect found in this study is likely directionally robust, but its precise magnitude may be overestimated due to shared method variance. Future research should employ multi-source (e.g., supervisor-rated IWB) and multi-wave designs to provide more definitive evidence on the discriminant validity between PC and IWB.

Our integration of the JD-R model and trait activation theory provides a powerful framework for understanding. However, some directions need to be explored: (1) Longitudinal studies and design: Future studies may use experimental interventions or multi-year panel data to prove causality. Such designs could help clarify whether AI enhances mental capabilities or builds upon pre-existing resilience traits. (2) Cross-cultural comparisons: Studying how social norms (e.g., power distance, uncertainty avoidance) affect error management culture can reveal cultural changes in AI influence. For example, leadership endorsement of AI may have disproportionately affected psychological capital in cultures where power is far more distant than in egalitarian cultures. (3) Specific technology mechanisms: Detailed analysis of AI sub-types (e.g., machine learning, robotics) can help to determine which technologies can work best in activating PsyCap or reducing innovation barriers. Comparative studies can also investigate whether employees perceive certain forms of AI as “collaborative” or “controlled.”

These limitations and directions highlight the need for context-sensitive research on human–AI interactions. By addressing these gaps, future research can help organizations harness the potential of AI while ensuring employee well-being and innovation.

## Data Availability

The raw data supporting the conclusions of this article will be made available by the authors, without undue reservation.
